# Supplementary material for: Are carbon emissions trading and green financial instruments synergistic? -Comprehensive quantitative research based on content analysis
Source: PLoS One. 2024 Mar 7;19(3):e0298601. doi: 10.1371/journal.pone.0298601 (PMC10919601; doi:10.1371/journal.pone.0298601)
Supplement: S1 Data — (ZIP) [file pone.0298601.s001.zip › Data/地级市Z_F工作报告.zip!/┴╔─■/╕╖╨┬╩╨/└┤╫╘╣1⁄2╓┌║┼í╛┬φ┐╦╩2╛▌═°í┐_2019.docx]

各位代表：

　　现在，我代表市政府向大会报告工作，请予审议，并请市政协委员和其他列席人员提出意见。

　　一、2018 年工作回顾

　　2018年是贯彻落实党的十九大精神的开局之年，是改革开放40周年。我们坚持以习近平新时代中国特色社会主义思想为指导，全面贯彻党的十九大和十九届二中、三中全会精神，认真学习贯彻习近平总书记在辽宁考察时和在深入推进东北振兴座谈会上重要讲话精神，坚持稳中求进工作总基调，深入落实新发展理念和“四个着力”“三个推进”，按照高质量发展要求，大力实施“突破辽西北”和县域经济发展战略，扎实开展“重实干、强执行、抓落实”专项行动，在省委、省政府和市委的坚强领导下，在市人大依法监督和市政协民主监督下，迎难而上，奋力攻坚，经济发展提质增速，社会事业全面进步，转型振兴迈出坚实步伐。

　　预计实现地区生产总值450亿元，增长6.6%；一般公共预算收入42 亿元，增长10%；固定资产投资132亿元，增长20%；城镇居民人均可支配收入27630 元，增长7.5%；农村居民人均可支配收入13460元，增长7.3%；城镇新增就业1.5万人，登记失业率4.8%。主要经济指标增幅高于全省平均水平，预计省政府考核的“突破辽西北”9 项年度指标全部完成。

　　第一，项目建设取得突破。全面完成“3215项目牵动计划”。固定资产投资增幅全省第一。总投资245亿元的大唐煤制天然气项目全面复工，投资35亿元的中新能化化工项目启动建设；页岩气陆相示范区列入国家规划，阜页1井压裂产气；上海富国整体搬迁完成。义乌小商品城、中仑新能源、彰武永红机械等投资五千万元以上的155个项目开工建设；德尔二期、省环保集团油泥处理等112 个项目加快建设；万达动车铸铝件、鲁花扩能、伊利二期等50 个项目竣工投产；新增亿元以上储备项目208个。

　　第二，工业经济企稳向好。工业运行摆脱多年低迷状态，在弥补去产能减量基础上，规上工业增加值增幅达到10.5%，税收增长12.2%。农产品加工、装备制造及配套、能源、化工四个优势产业总产值占全市工业75%。新能源产业快速发展，风电、光伏总装机容量达到236万千瓦，占全省总量的30%。新希望六和、小东北等一批企业实施技改扩能，远东铜业、力昌铸造等一批企业成为新的增长点。10个开发区初步建立“管委会+公司”管理模式，新入驻企业88户，彰武开发区、氟产业开发区集聚能力明显提升。液压装备特色产业基地通过国家复核，皮革开发区成为国家外贸出口转型升级基地。

　　第三，乡村振兴扎实推进。出台并实施乡村振兴战略实施意见，大力实施县域经济发展战略，阜彰两县在全省实现晋级升位。新增设施农业和高效农业15.6万亩，新建、改扩建畜牧养殖小区17个，优质种养业增产增效，实名制劳动力转移就业20.7万人，实现大旱之年农业不减收。全市“三品一标”认证产品达到157个。新型农业经营主体超过7000家，农村电商110家。宜居乡村建设扎实推进，8个特色小镇规划建设稳步实施。非洲猪瘟疫情防控有力。“大棚房”问题得到有效整治。成功创建全省唯一的“全国平安农机示范市”，阜蒙县现代农业产业园被列入省级产业园，彰武县被列为国家农村一二三产业融合发展先导区。

　　第四，服务业加快发展。实施消费升级行动计划，预计全社会消费品零售总额增长3.5%以上，限额以上单位网上零售额增长248%，快递业务量增长32%。6个省级服务业集聚区规模壮大，万达广场开业，居然之家等商业综合体建成，城市北部新商圈初步形成。文化旅游产业发展步伐加快，预热高铁经济取得良好成效，成功举办玛瑙文化旅游节，温泉城和黄家沟成为旅游新热点，百年赛道小镇启动建设，彰武草原生态恢复示范工程顺利推进。康养产业取得突破，北京博奥精准医学及健康产业项目正式落户，阜外医院心脏康复协作基地等项目达成意向。现代物流业初具规模，东北煤炭物流中心开工建设，意通物流园具备运营条件。全市运营电商园区达5个，互联网+、大数据等新兴服务业发展势头良好。金融机构新增贷款73亿元，增长7.9%。新引进金融机构2 家，金控公司试运行。

　　第五，深化改革释放活力。供给侧结构性改革扎实推进，低端产业逐步退出，中高端产业稳步成长。“三去一降一补”任务如期完成。商品住宅库存去化周期缩短到17.3 个月；采取建立工业水源、推行供电双边交易等措施，要素成本逐步降低。“放管服”改革深入实施，取消调整一批行政审批事项，制度成本有效降低。“互联网+政务服务”平台投入使用。“证照分离”“多证合一”改革启动实施。新增市场主体2万户，增长37%。营商环境得到优化，开展“办事难”“纠四风”专项整治，共取消调整各类证明253 项，8890政务便民服务平台运行，一般性经营企业开办时间压缩到3个工作日，制定《优化营商环境条例》。全面完成市政府机构改革任务，事业单位整合精简比例达到90%，经营性事业单位全部转企改制。国资国企改革稳步推进，交投集团率先进行混合所有制改革，生态、阜勤、农牧3个企业集团挂牌运营；“三供一业”分离移交完成，改造工程进展顺利；厂办大集体改革启动实施。出台27 条支持民营经济发展政策，“个转企”512户，“小升规”34户，民营经济占全市经济的75% 以上。市区财税、县乡财政体制改革完成，启动城区街道财政体制改革试点。

　　第六，开放合作步伐加快。积极参与“一带一路”建设，国际贸易快速增长，辽宁工大与俄罗斯等国家的高校、科研机构及企业合作扩大，阜新成为中蒙俄、辽蒙欧两大通道上的重要节点城市。加快融入沈阳经济区和京津冀协同发展，与江苏盐城合作共建环保产业园，与新疆八师石河子市合作领域不断拓宽。阜新国家高新技术产业开发区纳入辽宁自贸试验区协同发展序列，落地复制上海等自贸试验区改革创新措施21 项。狠抓“双招双引”，三河新材料等88个项目签约落地，内资到位85亿元。大力发展“飞地经济”，乡镇引进项目55个。成功举办农产品加工、硅砂、煤层气等高峰论坛。新增出口企业14户，外贸出口总额19 亿元，增长21.8%，增幅居全省第二。科技创新扎实推进，新增高新技术企业16 户。与辽宁工大共建科技创新产业园，20户企业加入产学研合作联盟。引进高学历人才200 人，新增院士和专家工作站11家。

　　第七，城乡环境明显改善。“三大通道”初步建成。京沈客专正式通车，阜新进入高铁时代；巴新铁路有效运行，成为重要物流通道；阜新至扎鲁特超高压输变电工程投入使用，破解了新能源消纳困局。锦阜高铁路扩能工程全面完成。改造国省干线103公里，新建改造农村公路644公里。维修改造18条城市破损道路、21条小街小巷。市容环境综合整治成效明显，拆除违建21万平方米，清除一批城市“顽疾”。棚改腾空地利用率达到60%。城市防洪“1+3”工程基本建成，佛寺水库除险加固工程完工。植树造林12.7万亩，玉龙山绿化工程启动。农村环境整治初见成效，农村改厕7033座，实施农村生活垃圾分类减量试点。中央环保督察整改工作深入推进，环保督察“回头看”新交办案件办结率达99.6%，全社会生态环保理念整体提升。开展采煤沉陷区基本情况普查，深入开展海州露天矿综合治理。完成国家第三次国土调查试点任务。污染防治攻坚战成效明显，市县乡三级“河长制”全面落实，完成黑臭水体整治任务；土壤污染防治工作全面加强，固体废物和危险废物实现有效处置；拆除燃煤小锅炉136台，淘汰黄标车及老旧车辆541辆，秸秆综合利用率达到90.7%，完成污染物减排年度任务，环境空气质量达标天数297天，位居全省前列。

　　第八，民生福祉稳步提升。民生支出占财政总支出达到83.3%。20件民生实事全部兑现。红光路打通，解决了周围居民多年的“出行难”；锦阜公路吕家店危险路段拓宽改造，消除全市最大的交通安全隐患；改造老旧小区29个，启动早期棚改小区维修工程；辽宁工大北校区学生宿舍采暖问题得到解决；人民公园健身步道翻新改造等等，解决了一批群众堵心事，做成了一批群众暖心事。脱贫攻坚任务如期完成，通过“五个一批”，16266 名贫困人口脱贫、48 个贫困村“销号”、阜蒙县脱贫“摘帽”。出台精准医疗扶贫方案，因病致贫返贫问题得到有效解决。基本医疗保障水平进一步提高，基层卫生服务能级全面提升。阜蒙县被国家民族蒙药协会认定为“蒙医药发祥地”。各县区一次性通过国家义务教育基本均衡认定，改造薄弱学校17所，维修校舍33万平方米。我市运动员在省运会获金牌97枚，辽宁男篮后备人才培训基地在我市挂牌。率先在全省建成智慧公交，新能源公交车占比达到63%。“阳光餐饮”工程启动，食品安全实现智慧监控。防范风险攻坚战扎实推进，政府债务、金融风险总体可控，养老金按时足额发放。扎实推进平安阜新建设，社会治安防控体系日益完善，扫黑除恶专项斗争深入开展。安全生产形势总体平稳。各类信访问题得到有效化解。民族团结进步事业不断发展，宗教领域和谐稳定。第四次全国经济普查全面铺开。国家卫生城创建通过省阶段性评审，国家文明城、双拥模范城创建有序推进。

　　第九，“重强抓”行动成效显著。以“重实干、强执行、抓落实”专项行动为总抓手，时间表、任务书、路线图“三位一体”，挂图作战，绩效对账，强力推进。坚持一级带着一级干，一级做给一级看，年初确定的255项重点任务全面落实，干部作风持续好转，行政效能明显提升。

　　政府自身建设全面加强。认真履行全面从严治党主体责任，坚持把政治建设放在首位，严守政治纪律和政治规矩，强化“四个意识”，坚定“四个自信”，践行“两个维护”，铸牢忠诚“第一底色”。严格落实“一岗双责”，充分运用监督执纪第一种形态，逐级压实党风廉政建设责任，全面落实意识形态责任制，严肃党内政治生活，扎实推进“两学一做”学习教育常态化制度化。狠抓中央和省委巡视反馈意见整改落实，持续修复净化政治生态。深入开展解放思想推动高质量发展大讨论，有效推动改革再深入、实践再创新、工作再抓实。加强法治政府建设，切实完善依法科学民主决策机制，加强审计监督，深入实施“七五”普法规划，依法行政水平持续提高。自觉接受市人大法律监督、工作监督和市政协民主监督，办结人大代表建议255件，政协委员提案199件。

　　全市各行各业、各条战线都呈现出新变化，外事、侨务、对台、国防动员、市志、档案、工会、青年、妇女儿童、红十字、老龄、慈善、残疾人保障等各项工作都取得了新成绩，税务、电力、邮政、通信、消防、气象、石油、烟草等单位为地方经济社会发展作出了积极贡献。

　　过去的一年，困难要比预想的多，结果要比预期的好。我们创新举措，真抓实干，干成了一批打基础利长远的实事难事，破除了一批存在多年的难点堵点，解决了一批百姓关心关注的身边事具体事。成绩来之不易，这是党中央、国务院亲切关怀和省委、省政府坚强领导的结果，是市委总揽全局、协调各方的结果，是全市人民奋发有为、合力攻坚的结果。在此，我代表市政府，向全市广大干部群众，向人大代表、政协委员和各民主党派、工商联、人民团体、无党派人士，向离退休老同志，向驻阜中省直单位、部队指战员、公安干警、武警官兵、消防救援队伍指战员，向所有参与、关心和支持阜新发展的同志们、朋友们，表示衷心的感谢和崇高的敬意！

　　同时，我们也清醒地认识到，对照高质量发展要求，阜新经济社会发展还存在许多困难和问题。主要表现在：经济总量小，严重制约城市发展；传统煤炭产业加速退出，接续替代产业尚未形成有效支撑；财政收支矛盾突出，政府偿债压力较大；民生领域短板凸显，养老金发放压力持续加大，保工资、保运转、保民生支出缺口较大；教育、医疗等社会事业领域的一些问题亟待解决；县域经济、城区经济发展相对滞后；营商环境仍需改善，办事难现象仍然存在，企业融资难、融资贵等问题尚未得到有效解决，各种要素成本还处于较高水平；一些政府工作人员思想解放不够，干事创业激情不足，专业能力和专业精神有待提升，不担当不作为问题仍然存在。对于这些问题，我们一定采取有力措施，认真加以解决。

　　二、2019年工作安排

　　今年是新中国成立70周年，是全面建成小康社会、推进阜新转型振兴的关键之年。我们要始终牢记习近平总书记的谆谆教导、殷切期望，切实把习近平总书记重要讲话精神转化为奋发昂扬的精神状态，细化为务实管用的思路举措，实化为真抓实干的具体行动，苦干实干会干，撸起袖子加油干，全力推进新时代阜新转型振兴。

　　今年政府工作的总体要求是：以习近平新时代中国特色社会主义思想为指导，深入贯彻党的十九大和十九届二中、三中全会精神，全面贯彻中央经济工作会议精神和习近平总书记在深入推进东北振兴座谈会上重要讲话精神，深入落实省委经济工作会议和市委十二届十一次全会暨市委经济工作会议精神，统筹推进“五位一体”总体布局，协调推进“四个全面”战略布局，坚持稳中求进工作总基调，坚持新发展理念和“四个着力”“三个推进”，坚持高质量发展，贯彻“巩固、增强、提升、畅通”八字方针，深化供给侧结构性改革，聚焦补齐“四个短板”，全力做好“六项重点工作”，深入实施“突破辽西北”和县域经济发展战略，继续开展“重实干、强执行、抓落实”专项行动，进一步稳就业、稳外贸、稳投资、稳金融、稳外资、稳预期，扎实推进阜新转型振兴，为全面建成小康社会奠定决胜基础，以优异成绩迎接中华人民共和国成立70周年。

　　主要指标预期是：地区生产总值增长6.5%，一般公共预算收入增长7%，固定资产投资增长15%，城乡居民收入增长与经济增长同步，全面完成“突破辽西北”和县域经济发展省考核指标。实现这些目标，我们要重点抓好以下九个方面工作。

　　（一）狠抓深化改革

　　持续优化营商环境。全面实施省市优化营商环境条例，继续实行领导干部帮扶企业制度，建立企业家日常联系机制，实现规上企业“管家”全覆盖。继续深化“放管服”改革，推行“证照分离”“多证合一”，进一步梳理行政职权，能减尽减、能放尽放。深化集中行政审批权改革，优化审批流程，缩短审批时间，努力打造全省审批效率最高的城市。改进“双随机、一公开”监管，减少对企业的各类检查，推进跨部门联合检查，必须进行的检查要严格报批。全面推进“一网、一门、一次”改革，行政审批和公共服务事项网上办理实现80%以上，“一门”实现应进必进，100个高频事项实现最多跑一次。巩固政府机构改革和事业单位改革成果，释放改革红利。

　　深化供给侧结构性改革。着力在“巩固、增强、提升、畅通”上下功夫。巩固煤炭去产能、清理“地条钢”成果，全力支持去产能退出企业转型发展；深入开展“五矿”共治专项行动，加大非煤矿山治理力度；全年房地产去库存30万平方米以上，建立促进房地产业健康发展的长效机制；综合施策，进一步降低实体经济各类成本，发挥企业和企业家能动性，增强微观主体活力；以创新驱动为引领，加快培育中高端产业集群，提升产业链水平；以持续扩大消费带动生产发展、以金融支持实体经济发展，以扩大就业为目标，畅通经济循环。

　　深化国资国企改革。加快推进国资监管由管资产向管资本转变，提高现有企业集团经营效益，组建水务集团。支持供热公司重组，推进混合所有制改革。加快建立法人治理结构，切实推行薪酬、人事、分配三项制度改革。全面完成“三供一业”维修改造。完成厂办大集体改革。深化市矿协作，推动市矿协同发展。

　　深化财政体制改革。巩固县乡财政体制改革成果，完善市区财税体制改革，全面推开城区街道财政体制改革，壮大乡镇经济、街道经济、村集体经济。

　　深化园区改革。全面实行“管委会+公司”管理模式，完善用人机制、薪酬制度和激励政策。把园区经济管理权限落实到位。复制辽宁、上海等自贸试验区制度创新成果。完善各园区发展规划，明晰产业定位、细化空间布局。完善基础设施，增强承载能力，促进园区快速发展。

　　（二）狠抓开放合作

　　深度融入共建“一带一路”。支持巴新铁路建设，推进阜奈高速尽早开工，加快聚宝源中欧创意园建设，积极融入中蒙俄对外开放大通道。主动参与省“一带一路”综合试验区、中东欧“16+1”示范区等重大平台建设。鼓励阜新企业到境外投资，参与国际市场竞争。

　　扩大外贸规模。做好稳外贸工作，巩固日韩俄等传统市场，精心组织参加进博会、广交会等重点展会，积极开拓国际市场。搭建外贸出口平台，实施外贸企业扩户工程，全年新增出口企业10户。

　　加强区域合作。积极参与京津冀协同发展，融入首都“两小时经济圈”、沈阳“一小时经济圈”，加强产业协作，承接产业转移。深化与江苏盐城合作，在特色产业、资源共享、科技交流、共建环保产业园等方面取得新成果。继续与新疆八师石河子市开展合作，做好对口援疆工作。

　　（三）狠抓结构调整

　　加快现代工业体系建设。推进传统工业转型升级，做优做大四个优势产业，实施富国皮革、远东铜业、力昌铸造等30个重大技改扩能项目。培育壮大新兴产业，全力抓好富新超纤革、德尔氢能源产业园、辽宁工大科技创新园、环保产业园等项目和园区建设。开展个转企、小升规、规升巨培育行动，年内至少完成个转企400户、新增规上企业40户、新增销售收入超亿元企业5户，规上工业增加值增长8%以上。

　　加快发展现代服务业。高水平编制完成高铁经济发展规划和全域旅游规划。建设旅游大通道，抓好温泉康养小镇、百年赛道小镇、彰武草原生态恢复示范区等项目规划建设。积极引进医养结合、养老服务等企业，加快发展康养产业。大力发展电子商务，促进线上线下融合发展，支持华唐大数据、四合大数据等平台建设。大力发展金融业，全力推进阜新银行上市，支持农信社改制，实现金控公司规范运营，引进金融机构2家。大力发展现代物流业，加快东北煤炭物流中心和义乌小商品城建设，意通物流园投入运营。全面开展扩大消费行动，举办玛瑙文化旅游节、篮球节、汽摩比赛等节庆、赛事活动，促进餐饮、住宿、商贸流通等传统服务业升级。

　　加快发展城区经济。把城区经济工作摆在更加重要的位置，坚持产业集聚、错位发展，科学确定各个城区产业定位。坚持工业牵动，实施争创省级开发区行动计划，全力以赴抓招商、上项目，提升工业经济实力。坚持服务业拉动，因地制宜打造特色街区，推动服务业集聚区提档升级。完善考评激励机制，促进城区经济高质量发展。

　　毫不动摇支持民营经济发展。全面落实国家、省、市支持民营经济发展的系列政策，鼓励民营经济拓展融资、扩大投资、创新发展。加强融资担保及再担保体系建设，坚持“政银企”定期例会制度，着力破解民营企业融资难、融资贵问题。执行市场准入负面清单制度，支持民营企业参与交通、水利、市政等公共事业投资运营，参与政府采购竞争。加快构建“亲”“清”新型政商关系，依法保护民营企业家人身财产安全，为民营经济发展创造健康环境。弘扬企业家精神，发挥企业家作用，聚焦实业、做精主业，不断提升我市民营企业核心竞争力。

　　实施创新驱动战略。围绕产业链部署创新链，深化与辽宁工大、中科院上海有机所等高校院所合作，鼓励企业加入产学研用联盟，加强众创空间、科技企业孵化器等平台建设，全年科技成果转化20项以上，科技对经济增长贡献率达到53%以上。鼓励大众创业、万众创新，培育更多市场主体。

　　（四）狠抓有效投资

　　突出抓好重大项目。继续开展“项目年”活动，推动三河新材料、50万千瓦光伏领跑者、德尔氢燃料电池、大金重工提质扩模、力达二期、中正钒钛材料等160个投资五千万元以上项目开工建设；推动中新能化化工、新邱独立工矿区改造搬迁、氟托二期、原本食品肉类加工等200个项目加快建设；推动大唐煤制天然气一期、中褚钛业、国华电力玍力马风电、昌盛日电光伏等100 个项目竣工投产。加快推进阜盘客专、巴新铁路到盘锦港延伸线等一批重大基础设施项目前期工作，包装储备投资亿元以上项目200个。

　　突出抓好“双招双引”。坚持精准招商、精选招商，加强专业招商、产业链招商、以商招商，借助重大展会、“辽商回归”平台，在招大引强上下功夫，全年引进内资100亿元。推动新兴铸管产业园、骆驼电池、今麦郎食品等项目签约落地。改进招商考核方式，注重结果导向，提高项目落地开工率。强化招才引智，重视引进领军人才，带动高端产业发展。抓好人才“引育用留”，继续引进高学历人才200名。

　　突出抓好“飞地经济”。出台支持“飞地经济”发展意见，建立利益分配和统筹协调机制。在现有园区规划一定区域，承接飞入项目。选择具备条件的乡镇规划建设飞地园区，年内每个乡镇街道至少引进一个飞地项目。

　　突出抓好服务保障。对投资2000万元以上项目配备“管家”，实行一个项目、一个领导、一个团队，提供“店小二”式服务。及时帮助协调解决项目建设中遇到的问题，全力保障项目顺利实施。

　　（五）狠抓乡村振兴

　　加快发展现代农业。出台我市乡村振兴战略规划。扎实推进农业供给侧结构性改革，保障粮食安全，优化“粮经饲”三元结构，发展草食性畜牧业，推动农牧复合发展。巩固现代农业示范带建设成果，启动15 万亩精品示范区建设，年内完成6万亩。积极开展“互联网+高质量农产品行动”，增加优质绿色农产品供给，新增“三品一标”认证产品11个。依托农产品产销联盟，扩大外销规模，叫响“阜新品牌”。推动农业“种养加运销”全链条发展，促进三次产业融合。大力发展民宿经济，形成乡村旅游新热点。继续做好非洲猪瘟防控工作。巩固“大棚房”整治成果，确保农地姓农。

　　加快发展县域经济。坚持以富民强县、晋级升位为目标，实施好三年行动计划，着力推进农产品加工、氟化工、铸造、硅砂等重点产业发展。统筹推进农产品生产基地、工业园区、服务业集聚区建设，支持彰武县争创省级现代农业产业园。突出地域特色，发展“一乡一业”“一村一品”，培育优势产业。

　　加强宜居乡村建设。继续做好十家子镇、章古台镇等8个特色小镇规划建设。学习浙江“千村示范、万村整治”工程经验，全面开展农村人居环境整治行动，重点抓好垃圾分类减量、畜禽粪污资源化利用、厕所革命、村容村貌提升，稳步提高农村卫生厕所普及率，农村生活垃圾处置体系覆盖75%的行政村。

　　深化农村各项改革。推进农村土地“三权分置”，放活农村土地经营权。做好农村闲置宅基地、集体建设用地及工矿废弃地综合利用工作。推进农村集体产权制度改革，让资源变资产、资金变股金、农民变股东。培育壮大农民合作社等新型经营主体，把小农户引入现代农业发展大格局。加强农村经纪人队伍建设，积极举办和参加各类展会，让优质农产品走向全国、走向世界。

　　（六）狠抓城乡建设

　　启动新一轮城市总体规划编制。优化城市空间布局，强化城市特色风貌规划，推行“多规合一”。按照突出特色、完善功能、错位发展原则，实现乡镇规划编制全覆盖。

　　加快高铁站前板块建设。开工建设高铁站前客运站，抓好学校、医院、公共服务设施等配套工程建设，提升集聚能力。实施“两路一街”延伸工程，建成龙湖路西段工程。完成玉龙山绿化工程，恢复旱河湿地，建设城市生态廊道。

　　加强基础设施建设。开工建设新希望大街、七家子镇至乌兰木图高铁站连接线、十家子镇至黑山高铁站连接线，完成南外环滑坡路段维修改造，实施彰武草原道路建设工程。启动101线部分路段拓宽改造，实现城市北环外扩，缓解市区交通压力。新建改造农村公路500公里，争创“四好农村路”示范市。实施柳河二期、绕阳河等防洪工程，完善城乡防洪体系。

　　提高城市管理水平。全面落实城市市容和环境卫生管理条例，实行网格化、精细化管理。继续开展城市环境综合整治，重点开展静态交通、市容市貌、违章建筑等专项治理，让城市环境更洁净、更宜居。以创建国家卫生城、文明城为载体，积极推进生态城市、海绵城市、智慧城市建设。加快维修改造小街小巷，做好城区绿化、亮化工作，增添城市活力。

　　（七）狠抓三大攻坚战

　　打好防范风险攻坚战。严控债务规模，规范发行地方专项债券，确保政府债务风险可控。做好排查预警，防范系统性区域性金融风险。严控养老金支付风险，优化支出结构、盘活存量资金、强化保费征缴，确保按时足额发放。

　　打好脱贫攻坚战。按照“两不愁三保障”要求，实施精准扶贫、精准脱贫，突出产业扶贫、就业扶贫、医疗扶贫，支持三产融合扶贫产业园区建设，实现所有建档立卡贫困人口全部脱贫，8个贫困村“销号”，彰武县脱贫“摘帽”，确保全面小康路上不落一户、不漏一人。

　　打好污染防治攻坚战。抓好海州露天矿综合治理规划、实施工作，创新新邱露天矿治理模式，打造国家绿色矿业发展示范区。做好采煤沉陷区普查工作。深入实施蓝天、碧水、青山、净土和农村环保五大工程，在“巩固气”的基础上“突破水”，确保细河高台子断面水质稳定达标。抓好中央环保督察和“回头看”反馈意见整改落实，确保整改到位。实施草原沙化防治、国土绿化等工程，筑牢生态屏障。严把新上项目环保关，实现环境保护和经济发展相得益彰。

　　（八）狠抓民生改善

　　着力提高就业质量和收入水平。坚持就业优先战略和积极就业政策，把稳就业放在更加突出位置，鼓励创业带动就业，实施高校毕业生就业创业促进计划和基层成长计划，引导高校毕业生留阜回阜就业创业。严格落实退役军人安置政策，大力促进退役军人就业创业。加强对去产能下岗职工、就业困难人员、贫困劳动力等重点群体的就业援助，发挥企业吸纳就业主渠道作用，全市城镇新增就业1.5万人，城镇登记失业率控制在5.2%以内。继续实施城乡居民和各类群体收入增长三年行动计划，确保城乡居民收入稳定增长。

　　持续提高社会保障水平。认真落实城乡低保、特困供养和退休人员基本养老金标准提高政策，改善孤儿和贫困残疾儿童群体保障条件。维护劳动者合法权益，做好农民工工资支付保障工作。提高医疗质量和医保服务水平，严厉打击医疗保险欺诈骗保行为。健全养老服务体系，让老年人安享幸福晚年。

　　优先发展教育事业。推动学前教育普惠健康发展，破解“入园难、入园贵”问题。切实加强教师队伍建设，严格规范中小学校办学行为，加快解决中小学生课业负担重、“大班额”问题。改善薄弱学校办学条件，提高高中和职教办学质量，促进教育均衡。

　　实施保障性安居工程。完成棚户区改造1266 套，维修早期棚改小区361 万平方米，改造居民室内供水设施5000户。加大农村危房改造力度，实现应改尽改。

　　保持社会和谐稳定。深入开展扫黑除恶专项斗争，保持对黑恶势力的严打高压态势，进一步夯实平安阜新建设成果。落实信访减存控增行动计划，及时解决群众合理诉求。全面压实安全生产责任，实行网格化管理，严防重特大事故发生。加强市场监管，积极创建国家食品安全示范城市。落实党的民族宗教政策，促进民族团结、宗教和谐。支持工会、共青团、妇联等群团组织开展工作。做好全国第四次经济普查、第三次国土调查工作，启动第七次全国人口普查。强化社会治理，巩固基层政权建设，做好村委会、居委会同步换届工作。进一步加强国防动员建设，做好双拥模范城创建工作。

　　今年，我们将办好海州桥翻建、主城区新增停车泊位3000个等20件民生实事，不断实现阜新人民对美好生活的向往。

　　（九）狠抓“重强抓”专项行动

　　坚持把“重实干、强执行、抓落实”专项行动作为推进工作、转变作风、塑造形象的总抓手，推动担当实干，保障各项工作落实落地。

　　聚焦目标重实干。对全年目标任务，进行细化分解，把责任落实到各级领导、各个县区、各个部门、落实到具体工作人员。建立对标机制，定期通报进展，鼓励先进，带动后进，确保完成全年目标任务。

　　聚焦重点强执行。紧扣重点难点，深入基层，加强调研，创新举措，精准发力，全力攻坚。坚持令出即行，一抓到底，做到不敷衍、不变通、不走样，确保干一件成一件、做一桩成一桩。

　　聚焦绩效抓落实。紧紧围绕省绩效考评指标、“突破辽西北”和县域经济发展考核指标体系，实行台账管理、定期对账。通过查作风、查协作、查责任，抓进度、抓质量、抓典型，推动各级各部门以更高的标准、更实的举措、更大的努力抓落实，真正干出实实在在的业绩。

　　各位代表，完成全年各项目标任务，关键在于抓好政府自身建设，打造人民满意政府。

　　加强政治建设。旗帜鲜明讲政治，树牢“四个意识”，坚定“四个自信”，坚决做到“两个维护”。严格履行全面从严治党主体责任，认真落实意识形态责任制，坚持以党建强队伍、以政治促业务，深入推进“两学一做”学习教育常态化制度化。

　　加强法治建设。提高政府干部运用法治思维和法治方式解决问题的能力，全面提升依法行政水平。做好地方性法规的实施工作。完善重大决策咨询、听证、合法性审查制度，保障科学决策、民主决策、依法决策。完善公共法律服务体系建设，加大普法力度，促进全民守法。自觉接受市人大法律监督、工作监督和市政协民主监督，认真办理人大代表和政协委员建议提案。

　　加强诚信建设。政府带头讲诚信，作出的部署必须落实到位，作出的承诺必须坚决兑现，新官必须理旧账。深入开展政府失信问题专项治理，严惩失信行为，以政府公信力取信于民。

　　加强廉政建设。坚持把党风廉政建设与中心工作一起谋划、一起部署、一起考核，认真落实“一岗双责”。聚焦行政审批、资源开发、工程招投标、脱贫攻坚等重点领域和关键环节，突出重点部门和岗位，加强廉政风险防控。加大审计监督力度，实行审计全覆盖。加强纪律教育，严格执行中央八项规定精神和省市委实施细则，严防顶风违纪和“四风”问题发生，坚决铲除滋生腐败土壤，促进干部清正、政府清廉、政治清明。

　　各位代表，转型振兴，使命光荣。让我们更加紧密地团结在以习近平同志为核心的党中央周围，高举习近平新时代中国特色社会主义思想伟大旗帜，在省委、省政府和市委的坚强领导下，怀着对阜新人民的炽热情怀，肩负转型振兴的历史重任，保持定力，坚定信心，真抓实干，以新气象新担当新作为奋力谱写新时代阜新经济高质量发展新篇章，以优异成绩向中华人民共和国成立70 周年献礼！
